# Hereditary angioedema due to C1 - inhibitor deficiency in Switzerland: clinical characteristics and therapeutic modalities within a cohort study

**DOI:** 10.1186/s13023-016-0423-1

**Published:** 2016-04-21

**Authors:** Urs C. Steiner, Christina Weber-Chrysochoou, Arthur Helbling, Kathrin Scherer, Peter Schmid Grendelmeier, Walter A. Wuillemin

**Affiliations:** Division of Clinical Immunology, University Hospital Zurich, Zurich, Switzerland; Allergy Unit, Department of Dermatology, University Hospital Zurich, Zurich, Switzerland; Division of Allergology, University Clinic of Rheumatology, Immunology and Allergology, Inselspital Bern, Bern, Switzerland; Allergy Unit, Department of Dermatology, University Hospital Basel, Basel, Switzerland; Division of Heamatology and Central Heamatology Laboratory, Department of Internal Medicine, Kantonsspital Lucerne and University of Berne, Berne, Switzerland

**Keywords:** Hereditary Angioedema (HAE), Gender related clinical characteristics, Danazol, Individualized therapy

## Abstract

**Background:**

Registration of trigger factors, prodromal symptoms, swelling localization, therapeutic behavior and gender-specific differences of the largest cohort of patients with hereditary angioedema due to C1-Inhibitor deficiency (C1-INH-HAE) in Switzerland.

**Methods:**

Questionnaire survey within a cohort study: Consenting eligible patients with diagnosed HAE according to clinical history, physical examination and laboratory results, including plasma values for C1-INH and C4 were selected. To each participant we sent a questionnaire assessing patients’ birthday, sex, date of first symptoms and diagnosis, trigger factors, prodromal symptoms, frequency and localization of angioedema, medication use and co-morbidities. Clinical information was collected in each center and then transmitted to the cohort database. Frequencies and distributions were summarized. Associations between gender and trigger factors or prodromal symptoms or localization of angioedema were assessed in multivariate analyses correcting for patients’ age.

**Results:**

Of 135 patients, data from 104 patients (77 %) were available for analysis. Fifty- four percent were female, mean age at diagnosis was 19.5 years (SD 14.1), Mean age when completing the questionnaire was 44.0 (SD 19.8). More women than men were symptomatic (44/57 vs. 36/47; *p* = 0.005). This association remained when correcting for age at diagnosis (16.10. 95%CI (5.17 to 26.70); *p* = 0.004). Swelling episodes ranged between 1 and 136 episodes/year. Swelling was more common among female than among male (-13.15 (95 % CI; -23.10 to -3.22), *p* = 0.010). Age at diagnosis was inversely associated with the total number of attacks 0.50 (-0.88 to -.011); *p* = 0.012). One third of patients were on danazol prophylaxis.

**Conclusion:**

We found large differences of HAE in male and female both in terms of symptom number and swelling episodes. Women are more affected by intensity and frequency of angioedema episodes than men. Danazol treatment remains widely used as effective prophylaxis despite its side effects. New therapies which selectively influence the hormonal estrogen balance could open new therapeutic options mainly for women and maybe also for men.

## Background

Hereditary angioedema with C1 inhibitor deficiency (C1-INH-HAE) is a rare, inherited disease, clinically characterized by recurrent acute swelling episodes on the extremities, abdomen, face, trunk, or airways, resulting from increased vascular permeability. These episodes occur spontaneously or are triggered by stimuli such as trauma, psychological stress, or infections. The swellings in HAE patients are highly variable in terms of trigger factors, severity, frequency and location [1, 2].

There are two types of C1-INH-HAE: Type I with decreased secretion of C1 inhibitor protein and Type II with secretion of a dysfunctional protein [3, 4]. Both result from an aberration in the gene coding for the plasma protein C1-inhibitor (C1-INH). Another type of HAE with normal C1-INH protein was first described in 1985. In some of these patients a mutation in F12 gene can be detected and they are classified FXII-HAE, the other patients without detection of a genetic defect are classified unknown HAE (U-HAE) [3–5]. According to several nationwide cohort studies the prevalence of HAE is estimated to be between 1:50000 and 1:100000 [6–9]. In Switzerland three studies describing the clinical characteristics of patients with HAE have been published so far [10–12]. Switzerland with approximately 8 million inhabitants is divided in four different language regions. The German part with about 70 % inhabitants, the French part (23 %), Italian part (6 %) and Rumantsch part (1 %). The present study is based on 135 patients living in the German part. Based on the estimated prevalence, the number of patients in our Swiss cohort corresponds to approximately 85 % of all HAE-affected patients living in Switzerland and thus represents the most complete set.

It is known that women suffer from more frequent and more severe angioedema attacks than men [13]. In this study, expanding on previous research, [13] we analyzed gender specific differences of HAE in more detail comparing trigger factors, prodromal symptoms, swelling localization and therapeutic behavior on women and men. Finally, we discuss treatment plans for different disease manifestations.

## Methods

The protocol of this study received Ethics approval and was conducted according to the principles of good clinical practice and strictly adhered to the ethical standards outlined in the Declaration of Helsinki [14].

### Identification of patients

Patients from the Division of Haematology of the Cantonal Hospital Lucerne, the Allergy Units of the University Hospitals from Zurich, Berne and Basel were included. All patients fulfilling the diagnostic guidelines criteria for HAE [3, 15] qualified for inclusion. All included patients gave their written informed consent. Patients from the French and Italian part of Switzerland were excluded because Ethics approval for this study was missing.

### Data collection

HAE was diagnosed on the basis of patient history, clinical examination and laboratory results, including plasma levels of functional C1-INH, antigenic C1-INH and complement factor C4. C1-INH-HAE type I was diagnosed when C4 was decreased and functional and antigenic C1-INH levels were below 50 % of normal. C1-INH HAE type II was diagnosed when C4 was decreased, functional C1-INH was below 50 % and antigenic C1-INH was above 50 % of normal. In 2013 a detailed questionnaire was sent to the participants, assessing the patients’ birthday, sex, date of first symptoms and diagnosis, trigger factors, prodromal symptoms, frequency and localization of angioedema, medication use and comorbidities. Patients answered the questions retrospectively for the year 2012. Specific additional clinical information was collected in a survey conducted in 2013 and 2014 via direct contact with the patient, the family physician, hospitals, or relatives. Because ethical approvals for detailed questionnaire of the centers of Zürich and Basel were not available, a closing evaluation of all data was possible for 104 patients of the centers Lucerne and Berne.

### Data handling

Clinical information was collected in each center and then transmitted to the administration site of the Swiss HAE cohort (Haematology of the Cantonal Hospital Lucerne). The information collected in the survey was screened and checked for plausibility by one researcher and then added to the electronic database. The cohort database is maintained by one researcher. If data were implausible or incomplete we contacted the patient or his / her caregiver for clarification. The final dataset was then pseudonymised and prepared for analysis. The pseudonymisation key is stored at the administration site.

### Statistical analysis

We summarized variates with means and standard deviations or percentages where appropriate. Associations between female gender and presence of prodromal symptoms (yes/no) were assessed in multivariate analyses correcting for patients’ age. The association between overall number of attacks and attacks on the abdomen were assessed using mixed linear models entering an indicator variate for the patient id as random factor. Also, we assessed whether the age at diagnosis was associated with the reported number of attacks. Analyses were performed using the Stata 14.1 statistics software package. (StataCorp. 2015. Stata Statistical Software: Release 14. College Station, TX: StataCorp LP.).

## Results

The analysis was based on 104 consenting patients (57 women and 47 men) with a mean age of 44.0 years (SD 19.8) who completed the questionnaire referring to 2012. Hundred and two patients were affected by C1-INH-HAE type I, 2 by type II.

The mean age at symptom onset was 11 years (SD 8.2). In men it was 9 years (SD 9.2) three years earlier than in women with 12 years (SD 7.6). For details please see Table 1. The average delay from the first symptoms until diagnosis in patients without a family history of the disease was 14 years (SD 11) for both women and men.

**Table 1:** Characteristics of 104 patients completing the questionnaire

Parameter	Frequency / mean (SD)
women / men	57 / 47
mean age (years)	44 (SD 19.8; range 5 - 80)
mean age women (years)	44 (SD 19.8; range 13 - 80)
mean age men (years)	41 (SD 19.7; range 5 – 72))
age groups	
≤10 years	3
11 – 20 years	12
21 – 30 years	17
31 – 40 years	14
41 – 50 years	18
51-60 years	21
≥60 years	19
symptom onset	
mean age symptom onset	11 (SD 8.2)
mean age symptom onset women	12 (SD 7.6)
mean age symptom onset men	9 (SD 9.2)
delay of diagnosis^a^	14 (SD 10.9)
delay of diagnosis^a^ women	n = 37: 14 (SD 10.3)
delay of diagnosis^a^ men	n = 21: 14 (SD 12.3)
HAE variants	
Type I	102
Type II	2

^**a**^affected people with unknown family history

*n* number

### Frequency and localization of angioedema

The gender-related frequency of angioedema is shown in Table 2. Women had more symptoms than men (45/57 vs. 33/47; *p* = 0.005) also when correcting for age at diagnosis (16.10. 95%CI (5.17 to 26.70); *p* = 0.004). Only 12 female and 14 male patients reported to be symptom free in the preceding year. Swelling episodes ranged between 1 and 136 episodes/year and was more common among female than among male (-13.15 (95 % CI; -23.10 to -3.22), *p* = 0.010). The localizations of angioedema occurred is shown in Table 3. Overall, attacks occurred most often on the abdomen (43 % cases), followed by the extremities, cerebral angioedema (headache), trunk, genitals, face and larynx. Age at diagnosis was inversely associated with the total number of attacks 0.50 (-0.88 to -.011); *p* = 0.012).

**Table 2:** Gender-related number of attacks of angioedema for 2012

	Total	Women	Men	
Patients	104	57	47	
symptomatic patients^a^	78	45	33	p = 0.563
number of angioedema attacks	1770	1290	480	p = 0.015
≥1/week^b^	10	8	2	
≤1/week; ≥ 1/month^c^	30	22	8	
≤1/month; ≥ 1/year^d^	38	15	23	

^a^Symptomatic: all patients who experienced angioedema in 2012; asymptomatic: all patients who didn’t experience angioedema in 2012

^b^frequent number of attacks

^c^intermediate number of attacks

^d^rare number of attacks

**Table 3:** Gender-related localization and number of angioedema in 2012

	women (57)		men (47)	
	n of patients	n of AE	n of patients	n of AE
abdomen	40 (70.%)	575	28 (59.%)	191
extremities	34 (60 %)	312	27 (57.%)	118
headache	11 (19 %)	186	5 (11 %)	43
trunk	15 (26.%)	89	7 (15 %)	37
genitals	11 (19 %)	70	11 (23.%)	35
face	14 (24.%)	41	8 (17 %)	33
larynx	9 (16 %)	17	7 (15 %)	23

Percentages are related in women to the total amount of women, in men to the total amount of men

*n* number

*AE* Angioedema

### Prodromal symptoms and trigger factors

Prodromal symptoms like fatigue, nausea, skin erythema, local paresthesia or itching, which may precede angioedema were reported by 51 % of women and 43 % of men (*p* = 0.397).

Eighty-one percent of female and 66 % of male respondents stated that trigger factors preceded angioedema attacks (Table 4).

**Table 4:** Trigger factors

Trigger factor	Number of patients	women	men
All	77	46	31
Emotions	53	33 (72 %)	20 (64 %)
Trauma / Mechanical trigger	47	27 (59 %)	20 (64 %)
Foodstuff / allergies	33	20 (43 %)	13 (42 %)
Infection	21	13 (28 %)	8 (26 %)
Hormones	35	34 (74 %)	1 (3 %)
None	27	11 (24 %)	16 (57 %)

Percentages relate to the total of women or men who noticed trigger factors

### Therapy

In Switzerland danazol and tranexamic acid (TA) are used for prophylactic treatment, whereas plasma - derived C1-Inhibitor (pd C1-INH) and Icatibant are administered for treatment of acute attacks. Some patients practice individual replacement therapy (IRT), which means that they administer pd C1-INH on demand very early when prodromal symptoms appear before an acute angioedema attack [16].

One third of patients were on prophylaxis. Danazol was used by 23 % of women and 27 % of men at doses of 100 mg twice a week to 200 mg daily. TA was taken by 10 % of women and 8 % of men with daily doses from 500 mg to 4000 mg. IRT was practiced by 7 women and 2 men.

In the event of an acute attack pd C1INH was administered to 56 % of women and 53 % of men, whereas 16 % of women and 6 % of men administered Icatibant. (Table 5).

**Table 5:** Therapeutic modalities in 2012 (*n* = 104)

Pharmacological prophylaxis	women and men	women	men
tranexamic acid	10	6 (10 %)	4 (8 %)
danazol	26	13 (23 %)	13 (27 %
IRT			
pd C1 - INH	9	7 (12 %)	2 (4 %)
Therapy of acute attacks			
Icatibant	12	9 (16 %)	3 (6 %)
pd C1 – INH	57	32 (56 %)	25 (53 %)

Percentages are related in women to the total amount of women, in men to the total amount of men

The frequency of angioedema episodes in 2012 with or without prophylaxis/IRT is shown in Table 6. Without prophylaxis, women suffered from 835 angioedema episodes (22/woman) and men from 216 (7/man). Women on danazol experienced 143 attacks (11/woman) and men on danazol 64 (5/man). Six women and 4 men using danazol had no attacks during the study period. Comparing gender-related localization of angioedema when on prophylactic treatment with danazol, women suffered from fewer symptoms on all locations, with the exception of the trunk, face and brain (headache) (Table 7).

**Table 6:** Frequency of attacks with or without prophylaxis / IRT in 2012

				Number of Angioedema attacks				
				no attacks	≥1/week^a^	≥1/month ≤ 1/week^b^	≥1/year ≤ 1/month^c^	Total n of AE
Women and men			104	26	10	30	38	1770
Women	All		57	12	8	22	15	1290
	prophylaxis	None	38	5	7	18	8	835
		Danazol	13	6	1	2	4	143
		TA	6	1	0	2	3	48
	IRT	C1-INH	7	0	2	4	1	264
men	All		47	14	2	8	23	480
	prophylaxis	None	30	9	1	5	15	216
		Danazol	13	4	0	2	7	64
		TA	4	1	1	1	1	78
	IRT	C1-INH	2	0	0	1	1	122

^a^frequent number of attacks; ^b^intermediate number of attacks; ^c^rare number of attacks

*n* number, *AE* Angioedema

no attacks: asymptomatic for the year 2012

**Table 7:** Prophylaxis with danazol and comparison of location-related symptoms between women and men in 2012

Location of attacks	All	Women			no prophylaxis vs. Danazol	Men			no prophylaxis vs. Danazol	women vs. men
		All	No prophylaxis	Danazol	*p*-values	All	No prophylaxis	Danazol	*p*-values	*p*-values
	104	57	31	13		47	28	13		
abdomen	68 (65 %)	40 (70 %)	24 (77 %)	6 (46 %)	p = 0.042	28 (59 %)	15 (53 %)	9 (69 %)	p = 0.344	p = 0.063
extremities	61 (59 %)	34 (60 %)	20 (64 %)	5 (38 %)	p = 0.111	27 (57 %)	14 (50 %)	8 (61 %)	p = 0.491	p = 0.157
trunk	22 (21 %)	15 (26 %)	8 (26 %)	3 (10 %)	p = 0.849*	7 (15 %)	4 (14 %)	0	p = 0.288*	p = 0.523*
face	22 (21 %)	14 (24 %)	8 (26 %)	2 (6 %)	p = 0.697*	8 (17 %)	4 (14 %)	1 (8 %)	p = 0.486**	p = 0.709**
genitals	22 (21 %)	11 (19 %)	8 (26 %)	1 (3 %)	p = 0.242*	11 (23 %)	5 (18 %)	3 (23 %)	p = 0.692*	p = 0.586*
headache	16 (15 %)	11 (19 %)	4 (13 %)	3 (10 %)	p = 0.404*	5 (11 %)	4 (14 %)	0	p = 0.288*	p = 0.509*
larynx	16 (15 %)	9 (16 %)	6 (19 %)	1 (3 %)	p = 0.654	7 (15 %)	4 (14 %)	1 (8 %)	p = 0.486**	p = 0.700**

* Fisher’s exact test, ** one-sided

Number of patients who suffered from at least one attack in the individual location in 2012

Among the patients on TA, one woman and one man were asymptomatic, one man suffered from frequent attacks (≥1/week) one man and two women from intermediate attacks (≤1/week; ≥ 1/month), and three women and one man from rare attacks (≤1/month; ≥ 1/year). Two women on IRT suffered from frequent attacks, four women and one man from intermediate, and one each from rare attacks.

Three different forms of treatment were practiced: Oral (Danazol, TA), intravenous (pd C1 INH), and subcutaneous (Icatibant). C1INH home therapy was practiced by 23 women and 15 men. The family doctor performed the therapy for 10 women and 5 men. Hospital treatment was required for 8 women and 4 men. Of the 30 patients who experience more than 1 attack/month, 16 were able to administer C1INH on their own at home. Two hundred and forty-eight swelling episodes were not treated by 21 women and 16 men.

### Comorbidities

The patients were asked about their medication intake other than HAE-related drugs. Specific medication allowed conclusion to be drawn about the diagnosis of other diseases than HAE. Thirty-two per cent, (20 women and 13 men) were affected by comorbidities (Table 8). Arterial hypertension was the most frequent disorder with 22 % (14 women, 9 men) followed by dyslipidemia at 5.7 % (5 women, 1 man). Patients with danazol-prophylaxis suffered with 54 % (9 women, 5 men) from more comorbidities compared to the 24 % (11 women, 8 men) without danazol prophylaxis.

**Table 8:** Comorbidities

Co-morbidities	All patients			Patients without danazol			Patients on danazol		
		w	m		w	m		w	m
All	104	57	47	78	44	34	26	13	13
No comorbidities	71 68 %	37 65 %	34 72 %	56 72 %	33 75 %	26 76 %	12 46 %	4 31 %	8 61 %
Comorbidities	33 32 %	20 35 %	13 28 %	19 24 %	11 25 %	8 23 %	14 54 %%	9 69 %	5 38 %
Arterial Hypertension	23 22 %	14 24 %	9 19 %	11 14 %	6 14 %	5 15 %	12 46 %	8 61 %	4 31
Dyslipidemia	6 6 %	5 9 %	1 2 %	0	0	0	6 23 %	5 38 %	1 2 %
DVT	4 4 %	1 2 %	3 6 %	2 2 %	0	2 6 %	2 8 %	1 2 %	1
Depression	4 4 %	2 3 %	2 4 %	2 2 %	1 2 %	1 3 %	2 8 %	1	1
CHD	4 4 %	3 5 %	1 2 %	4 5 %	3 7 %	1 3 %	0	0	0
CVI	1 0.9 %	0	1 2.1 %	0	0	0	1 3.8 %	0	1
PAD	1 1 %	0	1 2 %	1 1 %	0	1 2.9 %	0	0	0
Diabetes mellitus	5 5 %	4 7 %	1 2 %	3 4 %	2 4 %	1 3 %	2 8 %	2 15 %	0
Epilepsy	1 1 %	1 2 %	0	1 1 %	1 2 %	0	0	0	0
Schizophrenia	1 1 %	0	1 2 %	0	0	1 3 %	0	0	0
Parkinson’s disease	2 2 %	2 3 %	0	1 1 %	1 2 %	0	1 4 %	1	0
Hypothyroidism	2 2 %	1 2 %	1 2 %	1 %	1 2 %	0	1 4 %	0	1
COPD/Asthma	2 2 %	2 3 %	0	2 2 %	2 4 %	0	0	0	0
Osteoporosis	2 2 %	2 3 %	0	2 2 %	2 4 %	0	0	0	0

*CHD* Coronary heart disease, *CVI* Cerebrovascular Insult, *DVT* deep venous thrombosis, *PAD* peripheral artery disease

## Discussion

In this study we described the gender-related clinical and therapeutic characteristics of the great majority of Swiss HAE patients. Women were more affected by intensity and frequency of angioedema episodes than men, which is in line with a previous report [13]. This is most probably related to the exogenous and endogenous estrogen in puberty, ovulation during the menstrual cycle, estrogen-based birth control pills, or estrogen replacement therapy [17].

### Prodromal symptoms and trigger factors

About half of the patients, mainly women, noticed prodromal symptoms once in their lives. This awareness to perceive oncoming attacks enables the patients to start therapy early, which in turn leads to a quicker treatment response [18].

The proportion of patients reporting the perception of trigger factors corroborates the data recently described by Zotter and colleagues [19]. Mental stress, mechanical trauma and physical exertions are the most relevant trigger factors for both genders, followed by food and infections. Foodstuff as a trigger factor is also described by Zotter et al. If it is intolerance or allergy which induces angioedema has to be elucidated in further studies. In our study, a large variety of different foodstuff was reported, including milk products, fruit, seafood and nuts. Endogenous or exogenous estrogen is an exclusive and the most important trigger factor for women. Female were more prone to mental stress than men, physical and mechanical exertion were a more relevant trigger in men (Table 4). These results could indicate that women have a different coping with stress than men, whereas men are more exposed to physical/mechanical exertion. We speculate that women have a better self–perception enabling them to recognize prodromal symptoms and trigger factors better than men.

### Frequency and localization of angioedema

Regardless of gender, the abdomen was the most affected body part, followed by the extremities. In other cohorts this was the opposite [13, 20]. Compared to men, women suffered from more attacks to all localizations except the genitals and larynx. The constitutional exposure of genitals could explain why angioedema of this region occur more often in male. In order to identify the reasons for the more common laryngeal swellings in men, one can only guess that pharyngeal/laryngeal irritation by airway infections [19], snoring [21] or smoking [22] are relevant triggers.

### Gender related efficacy of danazol prophylaxis

Prophylactic danazol therapy is widely used in HAE-treatment. Its efficacy is well known, as are its side effects [23]. Danazol, on one hand, stimulates the liver to synthesize C1-INH and enhances the expression of C1-INH mRNA in peripheral blood mononuclear cells [24]. On the other hand, as a weak androgen, danazol has an antigonadotropic effect and inhibits ovarial estrogen production. If the daily dosage does not exceed 200 mg and treatment is closely monitored for possible side effects such as virilisation, hepatic diseases, arterial hypertension and dyslipidemia, it is a reasonable therapy for adult patients and those women who are neither pregnant nor breast-feeding [15, 25–29]. Development of liver cell adenomas or even hepatocellular carcinomas on long term therapy with danazol are uncommon [30, 31].

Comparing danazol prophylaxis between women and men, it seems that the drug has a better and different effect in women than in men.

Women on danazol suffered from fewer attacks to the abdomen and extremities than men on danazol (Table 7). It can be assumed that the hormonal modulating effect of danazol with suppression of gonadotropin and reduced estrogen production leads to a decreased stimulation of the contact activation pathway and consecutively to a decreased bradykinin production, which is essential in this prophylactic therapy. The high therapeutic efficacy of danazol in women could also explain why an important part of women (23 %) are on danazol prophylaxis despite a higher frequency of side effects.

### Comorbidities

In this cohort, patients on danazol suffer from more comorbidities (54 %) compared to those without prophylaxis (24 %). Women on danazol were more affected by comorbidities than men on danazol (69 % vs 38 %). Danazol treatment showed a clear association with Dyslipidemia.

### Therapy of attacks

Attacks were treated with pd C1-INH in 55 % of cases. Only few used Icatibant. The reason for this might be that Icatibant has not been on the Swiss market long enough (since 2009) and patients require a certain time to gain trust in new therapies. On the other hand, therapy with C1-INH seems to be more sustainable for patients with frequent attacks.

About two-thirds of patients using C1-INH practice home treatment after being carefully instructed in a HAE– center [32]. Offering home therapy and self-treatment to all HAE patients is a goal worth striving for. It’s the best way of optimizing treatment for a disorder with highly individual symptoms, improving quality of life with fewer and less severe attacks. As they do not require medical facilities, their medicare costs also decrease. Against doctors’ recommendation, 14 % of acute attacks were not treated at all. This is most likely because of an attitude that attacks that do not seem to be life-threatening do not require treatment.

## Conclusion

The aim in treating HAE-affected people is to minimize their swelling episodes in the best possible manner with the limited therapeutic options. First and foremost, the treating physician should be aware that symptoms vary across individuals and that women suffer from a more severe disease activity than men. The therapy plan must include careful training in self-perception to recognize prodromal symptoms and trigger factors and a training in administering self-therapy for acute swelling episodes. This should lead to lifestyle adaption by avoiding triggers as much as possible and individualizing the treatment with early start of therapy according to prodromal symptoms and acute attacks. This gives the patient independency and more self-responsibility. Danazol, as an efficient prophylaxis, requires close supervision of side effects. Women seem to have a better benefit from danazol than men, but also suffer from more side effects. But this assumption should be confirmed in a prospective study comparing genders and frequency of angioedema before and with danazol. New therapies which selectively influence the hormonal estrogen balance could open new therapeutic options mainly for women and maybe also for men.

## Abbreviations

HAE: hereditary angioedema
pdC1-INH: plasma derived C1-Inhibitor
FXII: factor XII
TA: tranexamic acid
